# Brooding and parthenogenesis enhance the success of the coral *Porites astreoides* relative to *Orbicella annularis*

**DOI:** 10.1002/ecy.70102

**Published:** 2025-05-08

**Authors:** Don R. Levitan, Kevin C. Olsen, Rachael M. Best, Peter J. Edmunds

**Affiliations:** ^1^ Department of Biological Science Florida State University Tallahassee Florida USA; ^2^ State Fisheries Genomics Lab, Coastal Oregon Marine Experiment Station, Department of Fisheries, Wildlife and Conservation Sciences Hatfield Marine Science Center, Oregon State University Newport Oregon USA; ^3^ Department of Biology California State University Northridge California USA

**Keywords:** asexual reproduction, clonal propagation, coral demography, coral genetic structure, coral reef decline, mating systems

## Abstract

The abundance of many Caribbean corals has declined over the past few decades, yet now *Porites astreoides* is more common on many shallow reefs than in the 1980s and shows evidence of local adaptation. We compare the small‐scale (1–8000 m) genetic structure of this brooding species and the broadcasting coral *Orbicella annularis* on reefs (<14 m depth) in St. John, US Virgin Islands, to examine how larval dispersal and asexual propagation contribute to the retention of genotypes within reefs. Populations of *P. astreoides* have genetic structure across reefs separated by a few 100 m, increased relatedness within reefs, and parthenogenetic larval propagation confirmed by parent–offspring genotyping. Within reefs, *P. astreoides* colonies <1 m apart are more related, independent of clonal reproduction, than corals at greater distances. In contrast, *O. annularis* lacks across‐reef genetic structure, has low relatedness within and among reefs, and does not produce asexual larvae. Small‐scale genetic structure and high relatedness in *P. astreoides* are evident even without considering asexual propagation, but asexual reproduction enhances these differences. Neither species shows the genetic signature of inbreeding or reduced genotypic diversity despite the high within‐site relatedness of *P. astreoides*. Monitoring on these reefs from 1987 indicates that *Porites* has increased in abundance while *Orbicella* has decreased in abundance. The success of *Porites* is due to greatly increased settlement and recruitment compared with *Orbicella*. Together these results indicate that high numbers of locally retained and successful genotypes might explain the relative success of *Porites* on shallow, present‐day reefs in the Caribbean.

## INTRODUCTION

Flowering plants and benthic invertebrates share a variety of dispersal, mating, and other life history traits. This is particularly the case with marine species that cast sperm into the environment, retain brooded eggs, and release larvae that can settle near their maternal parent (Olsen et al., 2020). These brooding species are often hermaphroditic, occasionally exhibit mixed populations of hermaphrodites and female‐only individuals, and show variation in the degree to which self‐fertilization is blocked (Olsen et al., 2021). A less common shared trait is the production of asexual parthenogenetic offspring (e.g., Dandelions; Molina‐Montenegro et al., 2013, Corals; Ayre & Miller, 2004). Parthenogenesis is distinct from asexual fragmentation and vegetative growth common in many clonal and colonial marine invertebrates and plants. Unlike vegetative propagation, parthenogenetic propagules have the same potential for dispersal as sexual propagules and the same potential for influencing population structure, nonrandom mating via inbreeding, and local retention or dispersal of genotypes. We studied *Porites astreoides*, a brooding parthenogenetic Caribbean coral, and the historically dominant but now declining *Orbicella annularis*, a broadcasting coral that releases eggs and sperm into the environment for external outcrossing fertilization and planktonic development. This contrast in life history sheds light on the mechanisms favoring *P. astreoides* becoming a dominant shallow‐water coral in the Caribbean following decades of anthropogenic disturbances.

Reef‐building corals have been declining in abundance throughout the world for decades (Tebbett et al., 2023), with this trend driven by multiple types of disturbances including thermal stress (Hughes et al., 2017) and disease (Rogers, 2009) that increase coral mortality and algal overgrowth, which limit settlement and recruitment of corals (Arnold et al., 2010). Analyses of long‐term coral abundances highlight interspecific differences in the rate and direction of change in coral cover (Toth et al., 2019). Demographic differences among species have motivated analyses of coral dynamics by functional groups (Darling et al., 2013). The emerging pattern is that the major reef‐building corals that are large, typically long‐lived, and reproduce via broadcast spawning have been decreasing in abundance to a greater extent (de Bakker et al., 2016; van Woesik et al., 2011) than smaller brooding species (Green et al., 2008; Toth et al., 2019).

The majority of corals presently recruiting to Caribbean reefs brood their eggs and release larvae (Edmunds, 2018; Gleason & Hofmann, 2011) while broadcasting coral recruits in this region are currently rare on settlement tiles and on natural reef surfaces (Arnold et al., 2010; Edmunds, 2018). The decadal‐scale increase in the relative abundance of brooding corals on Caribbean reefs is likely attributed to at least three factors. First, brooders are spermcasters and release sperm into the environment for collection by coral colonies for internal fertilization, which relaxes the need for highly synchronized spawning and reduces the likelihood of sperm limitation at lower population densities (Yund, 2000). Broadcasting requires a high degree of spawning synchrony and a high density of spawning individuals, as eggs and sperm can quickly dissipate and reduce the likelihood of fertilization (Levitan et al., 2004). As coral abundance declines, fertilization limitation should negatively affect broadcasting corals disproportionately. Second, brooding corals release larvae that are typically immediately competent to settle (Carlon & Olson, 1993), reducing time in the water column and increasing the likelihood of settling on nearby substrata. Broadcasting eggs develop in the water column, increasing the risk of planktonic mortality and of drifting to unsuitable habitats. Brooded larvae are larger, which can promote early growth and survivorship compared with smaller, less provisioned broadcasted larvae (Emlet & Hoegh‐Guldberg, 1997). Finally, brooding species often release larvae over many days over a lunar cycle, while broadcasters often spawn on very few evenings a year (Harrison et al., 1984). Combined, these traits might allow brooding species to be more successful at recruiting onto substrata that are unpredictably available in disturbed habitats (Goodbody‐Gringley, 2016). The costs of brooding include smaller adult size with typically lower adult survivorship, reduced fecundity, and reduced dispersal of larvae (Strathmann & Strathmann, 1982), notably away from relatives that might increase the risk of competition (Burgess et al., 2023) or inbreeding (Johnson & Woollacott, 2010; Olsen & Levitan, 2023).

A fourth trait, noted in a few brooding coral species, is the production of parthenogenetic larvae. This ability has only been reported in *P. astreoides* (Vollmer, 2018) and *P. divaricata* (Lord et al., 2023) in the Caribbean, and *Pocillopora damicornis* in the Indo‐Pacific (Ayre & Miller, 2004; Combosch & Vollmer, 2013). This capacity, along with the other traits associated with brooding, reflects a life history similar to parthenogenetic dandelion populations, which are highly successful in disturbed terrestrial habitats and show evidence of rapid local adaptation (Molina‐Montenegro et al., 2013). Parthenogenesis might be a key trait that explains why *P. astreoides* has increased in abundance on degraded reefs in the Caribbean, even compared with other brooding species (Green et al., 2008). *P. astreoides* has generally maintained absolute cover on shallow reefs where the overall coral cover has been declining, and therefore, its relative cover has increased from <10% in the 1970s to >40% in the 2000s (Green et al., 2008). At our study sites in Lameshur Bay, St. John, US Virgin Islands, demographic analyses indicate that *P. astreoides* often exhibits positive annual population growth (Edmunds, 2010) and has continued to increase in abundance of colonies (Edmunds et al., 2021), while the broadcasting *O. annularis* generally shows negative population growth (Edmunds, 2019).

We examine the mating system (degree of outcrossing, selfing, and parthenogenetic reproduction) and small‐scale (0.3–9.0 km) population genetic structure of *P. astreoides* at <6 m depth on Caribbean reefs to provide insight into why this species has been successful in the face of the general decline in abundance of Caribbean corals. We contrast our results for *P. astreoides* with the broadcasting coral, *O. annularis* (sampled at 9–14 m depth), which is declining in abundance throughout the region (Edmunds, 2015). Although previous studies have examined the population structure of these species (e.g., Foster et al., 2013; Riquet et al., 2022; Serrano et al., 2016; Severance & Karl, 2006; Shilling et al., 2023), our study compares the genetic structure of these coral species at sites where their populations have been studied annually since 1987. We confirm parthenogenetic larval reproduction in *P. astreoides* by comparing maternal and larval genotypes and quantify genetic isolation and relatedness of corals within and across these reefs, and the degree to which colony fragmentation and parthenogenetic reproduction contribute to genetic structure. We provide long‐term population data on these two species, at (or very close to) these sites, to examine the degree to which mortality and recruitment drive the changing patterns of abundance of the corals. The results suggest that the high levels of recruitment in *Porites*, but not *Orbicella*, might be facilitated by local larval retention of successful genotypes.

### Reproduction of study species

*P. astreoides* has a mating system consisting of simultaneous hermaphrodites, female colonies, and rare male colonies (Chornesky & Peters, 1987; Soong, 1991). In Jamaica, colonies <125 cm^2^ were more likely to be hermaphroditic, while colonies >250 cm^2^ were more likely to be female (Chornesky & Peters, 1987). The evidence for self‐fertilization in *P. astreoides* is equivocal, with the first genetic examination of brooded larvae, using RAPD markers, describing a ratio of 1/3 self‐fertilized to 2/3 outcrossed larvae released in Florida (Brazeau et al., 1998). Brazeau et al. (1998) excluded parthenogenetic reproduction, as larvae rarely shared identical amplification patterns with siblings or the maternal colony. More recently, an examination of mating systems in *P. astreoides* using microsatellite markers, also from colonies in Florida, found that 100% of the brooded larvae were parthenogenetically produced, with no signs of self‐fertilization or outcrossing (Vollmer, 2018). *P. astreoides* releases larvae monthly in the spring and summer in some locations, and year‐round in other locations, with individual colonies releasing larvae in batches over ~2–3 weeks leading up to the new moon (Chornesky & Peters, 1987; Soong, 1991; Vollmer, 2018). Laboratory studies indicate larvae are released at night and travel upward, but within a few hours descend to the benthos to explore the substratum, attach, and metamorphose (McGuire, 1997). Field observations of other brooding coral species indicate that larvae often settle within 10 min of release (Carlon & Olson, 1993).

*O. annularis* is a broadcasting hermaphrodite that spawns annually in the late summer or fall (Levitan et al., 2004, 2011). Each ripe polyp in the colony produces a single gamete bundle containing ~100 eggs packaged with millions of sperm (Szmant et al., 1997). Upon release, the bundle floats to the surface and dissipates into individual gametes (Levitan et al., 2004). Self‐fertilization has rarely been noted, and because outcrossed sperm is required for fertilization, there is no evidence to support the occurrence of parthenogenesis (Levitan et al., 2004; Szmant et al., 1997). Eggs of *Orbicella* develop into swimming planulae within 2 days of fertilization, and larvae are competent to settle 3–5 days after fertilization but can remain viable for up to 2 months in the laboratory (Miller et al., 2020).

## METHODS

### Genetic analysis and mating systems

**FIGURE 1:**
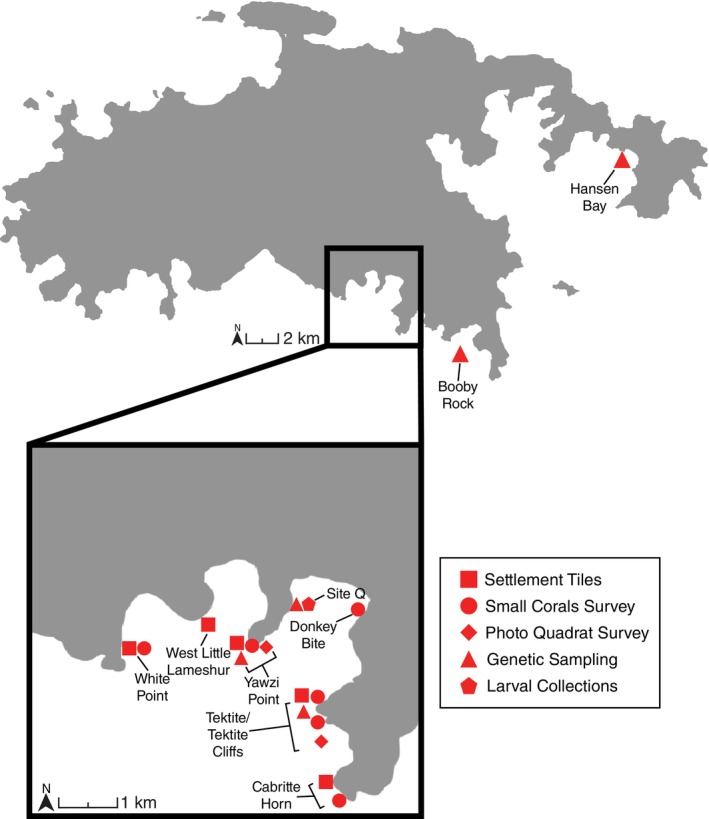
Map of St John, US Virgin Islands with insert of Lameshur Bay. Locations of genetic, population, recruitment, and settlement sampling. Site Q is where larvae were collected from maternal colonies.

Population structure, asexual propagation, relatedness, and inbreeding were examined for *P. astreoides* and *O. annularis* at four sites along the south coast of St. John, US Virgin Islands (Figure 1). Two sites in 2021, 0.4 km apart, were sampled in Lameshur Bay, Yawzi Point, and Tektite, and two sites in 2022 were sampled to the east: Booby Rock, 0.5 km offshore and 1.9 km east of Tektite, and Hansen Bay, 8 km east of Tektite. A fifth site (Site Q) was established 0.2–0.3 km north of the Yawzi Point site to collect and genotype brooded coral larvae from *P. astreoides* and sample maternal and nearby colonies for genetic analysis (Figure 1).

At each of the four survey sites, and for both species, a 50‐m transect was laid and every 5 m a coral and its nearest conspecific was sampled within the same habitat and depth (*n* = 20 corals/species/site). The yellow morph of *P. astreoides* was sampled at 3–5 m depth, and *O. annularis* was sampled at 9–14 m depth. For each colony, a tissue biopsy was taken with a 5‐mm‐diameter steel punch that was sterilized between cores, fixed with DNA/RNA Shield (Zymo Research) and stored at −20°C.

To examine mating systems in *P. astreoides*, in 2023, a 10 × 5 m quadrat, subdivided into 1‐m^2^ plots was established (Site Q). Within this site, 18 colonies were chosen (maternal colonies) to collect larvae as they were spawned using larval traps. Larval traps consisted of a mesh net that covered the entire coral with a buoyant detachable container on top (Appendix S1: Figure S1). Larval release occurs at night (McGuire, 1997) travel upward and get caught in the trap. Maternal colonies were chosen based on their size and orientation to facilitate a tight fit of the trap to ensure that only larvae from the chosen colony were collected. Traps were set each afternoon and collected the following morning, starting on 29 July for 13 days (full moon was on 31 July 2023). Individual larvae were collected and stored with 10 μL of DNA/RNA shield and frozen at −20°C. Site Q was subdivided into 1‐m^2^ plots, and the size (largest and perpendicular planar diameters), position, and tissue samples of these 18 maternal corals were collected along with any conspecifics within the 1‐m^2^ plot containing a maternal colony. For the 1‐m^2^ plots without maternal colonies, only conspecific location was noted to determine local density.

Adult tissue was digested with CTAB/proteinase K and DNA was extracted with phenol/chloroform. Individual larvae were added to a microcentrifuge tube with PCR buffer, Proteinase K and Tween 20 and digested in a thermocycler. Coral colonies and their larvae were genotyped using microsatellite loci. After an initial screening, based on Hardy–Weinberg equilibrium, linkage, and ease of scoring using Geneious Prime software, 8 loci for *P. astreoides* and 9 loci for *O. annularis* were selected (Appendix S1: Tables S1 and S2). In marker sets for both species, we evaluated the ability to distinguish clonal and non‐clonal members with genotype accumulation curves in the R (version 4.3.2) package “poppr” (Kamvar et al., 2014). For a given genetic dataset, these simulations quantify the power to delineate multi‐locus genotypes (MLG) within clonal species. The results indicated four and six loci were sufficient to identify clonal and non‐clonal members for *O. annularis* and *P. astreoides*, respectively (Appendix S1: Figure S2). We quantified the probability of obtaining the same MLG by common descent or chance with the P_sex_ statistic implemented in Genclone 2.0 (Arnaud‐Haond & Belkhir, 2007). The probability of two samples sharing the same MLG and being derived from sexual reproduction as opposed to cloning, was *p* < 0.0001 for *P. astreoides* and *p* < 0.0000001 for *O. annularis*. Given the results of genotype accumulation curves and these probabilities, we assigned individuals who shared the same MLG as “ramets” (clonemates) within the same “genet” (unique genetic individual).

We evaluated the ability to effectively estimate relatedness (*R*) from genotypic states (Appendix S1: Figure S3) using the number of loci, degree of polymorphism, and allele frequency to simulate parent–offspring, full sibling, half‐sibling, first cousin, and unrelated genotypes with the program Coancestry (Wang, 2011). The degree of relatedness among simulated genotypes was estimated with the triadic maximum likelihood estimator (TrioML). For each class of simulated relationship, we compared estimates of relatedness to values expected under random mating. Although these estimates have variance and any single pairwise estimate can be imprecise, our simulations tracked with expected values. Our analyses focused on mean estimates of relatedness, which tend to be more precise than single pairwise estimates (Olsen & Levitan, 2023). At site Q, where we compared maternal and larval genotypes and had finer scale sampling of *P. astreoides*, we examined the spatial autocorrelation in genotypic state among colonies with Moran's *I* using SPAGeDI (Hardy & Vekemans, 2002). We distinguished clonal and sexual reproduction by comparing the genotypes of maternal colonies and their offspring. Offspring with the same MLG as the maternal colony were characterized as being clonally produced, and offspring with non‐maternal alleles were characterized as sexually outcrossed. The probability of offspring being the product of sexual reproduction and sharing the same MLG as the maternal colony was assessed with the P_sex_ statistic.

### Population dynamics of *Porites* and *Orbicella*

The percent cover of corals was quantified at Yawzi Point (9 m depth) and Tektite (14 m depth) (Figure 1). Sites and methods are described in Edmunds (2024), but in brief, these reefs were quantified with photoquadrats (1 × 1 m) placed along three permanently marked transects (10 m) at each site. Ten quadrats were recorded along each transect, but annual sample sizes slightly varied due to field logistics. The photoquadrats were analyzed using a grid of 200 randomly located dots placed on each image, with the substratum category beneath each dot identified, summed, and expressed as a percentage of the dot population. We report the percentage cover of *Orbicella* spp. and *Porites* spp. from 1987 to 2022.

The abundance of early life stages of corals was quantified as the number of settlers (~2 mm diameter) and small corals (≤4 cm diameter) on settlement tiles and natural reef surfaces, respectively. Sites and methods are described in Edmunds (2018). In brief, coral settlers were quantified using settlement tiles (unglazed terracotta, 15 × 15 cm) secured horizontally in clusters of 15 tiles at each of five sites (Figure 1). The tiles were seasoned in seawater before deployment, immersed for a year (July–July), retrieved, bleached, and scored for the abundance of coral settlers using a microscope (40×). Corals were identified to family, and we report a decade of data (2015–2024) using sites as replicates within each year (*n* = 6). Poritidae include *Porites* spp., and Faviidae includes *Orbicella* spp. and *Favia* spp. Based on the abundance of adult corals, most faviid were probably *Favia fragum*, and thus the number of faviid settlers is likely to overestimate the density of *Orbicella* settlers. The abundance of small corals was recorded underwater using quadrats (0.5 × 0.5 m) placed at random locations along a permanently marked transect (40 m) at six sites (Figure 1), with five at 5 m depth and one at 9 m depth. Corals were identified to genus, and we report the number of *Porites* spp. and *Orbicella* spp. for 10 years (2014–2024 excluding 2023). At these same sites, a subset of small corals was tagged to assess annual mortality (methods in Edmunds, 2018) and we report the annual mortality for *Porites* spp. and *Orbicella* spp. for the last decade (2015–2024). Mortality data are pooled among the six sites to generate a robust estimate for Porites spp. (*n* = 370 colonies over a decade) and a weak estimate for *Orbicella* spp. (*n* = 21 colonies over a decade).

## RESULTS

### Asexual reproduction

Asexual propagation was prevalent in *P. astreoides*. At the four survey sites, the number of unique MLGs within a site ranged from 13 to 16 out of 20 colonies (average 71.21%, Appendix S1: Figure S4). At each site, three genotypes had clonemates among the 20 corals sampled. In some instances, clonemates were the focal individual's nearest neighbor, but in the majority of instances, clonemates were spread throughout the site and at times >40 m apart (Appendix S1: Figure S4). In a single case, clonemates were identified across two reefs separated by 5.7 km (Hansen Bay with one representative, and Booby Rock with two representatives). Sampling at Site Q had a much larger sample size (71 colonies) in a smaller area and found that the fraction of unique MLGs was 36.62% (Appendix S1: Figure S5).

Clonality in *O. annularis* was rare and restricted to the nearest neighbor (Appendix S1: Figure S6). Three pairs of clonemates were found, two at Tektite and one pair at Hansen Bay (proportion of MLG = 96.25%). Nearest conspecific neighbors at three sites were ≤2 m from the focal colony, and at Booby Rock, the density of this species was lower and the nearest conspecific neighbors were ≤6 m from the focal colony (Appendix S1: Figure S6).

### Population genetic structure

**FIGURE 2:**
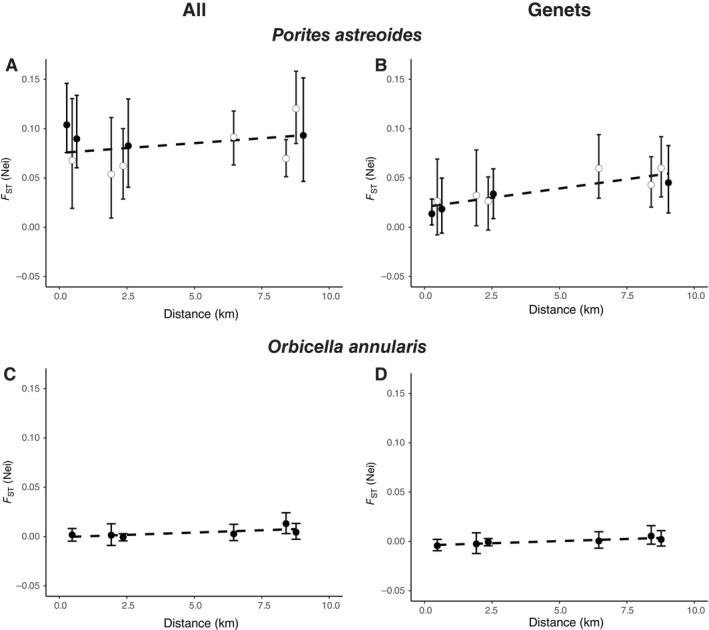
Population structure as a function of pairwise site distances for All colonies and Genets for *Porites astreoides* (A, B) and *Orbicella annularis* (C, D). Significant structure was only found in *P. astreoides*. Neither species showed significant isolation by distance. Open symbols for *P. astreoides* are the pairwise values that include Site Q used for larval collection. Error bars show 95% bootstrapped CIs.

Population genetic structure was analyzed with and without considering asexual propagation. Analyses with “All” individuals (using all ramets across all genets) considered the contribution of all colonies to patterns of population structure, while “Genets” are represented by a single instance for each MLG. Population structure in *P. astreoides* was significant among reefs in both datasets (Figure 2). Analysis of “All” individuals noted *F*_ST_ values significantly >0 (95% CI) for all pairwise comparisons, even between sites within Lameshur Bay separated by 0.27 km. Among the four survey sites where both species were sampled, the average pairwise *F*_ST_ was 0.078 in *P. astreoides*. In the “Genet” dataset, the level of *F*_ST_ was reduced to an average of 0.041. However, even with Genets, all sites >2.5 km apart had significant *F*_ST_ values, and two of the five sites separated by <2.5 km had significant *F*_ST_ values (including the two closest sites separated by 0.27 km within Lameshur Bay). Although there was a trend of increased isolation by distance, this was not significant (Mantel test, df = 1, correlation = 0.36, *p* = 0.22 for All and df = 1, correlation = 0.86, *p* = 0.07 for Genets) (Figure 2).

There was little evidence of population genetic structure in *O. annularis* with *F*_ST_ values of 0.004 for “All” individuals and 0.001 for “Genets” (Figure 2). Differentiation between one pair of sites, Tektite and Hansen Bay, had an *F*_ST_ value significantly greater than zero (*F*_ST_ = 0.013) in the analysis of “All” individuals, but this subtle structure was not present in the “Genet” analysis. Tektite and Hansen Bay were the only sites in which we detected fragmented genets, and this positive value of *F*_ST_ reflects the influence of fragmentation.

### Relatedness

**FIGURE 3:**
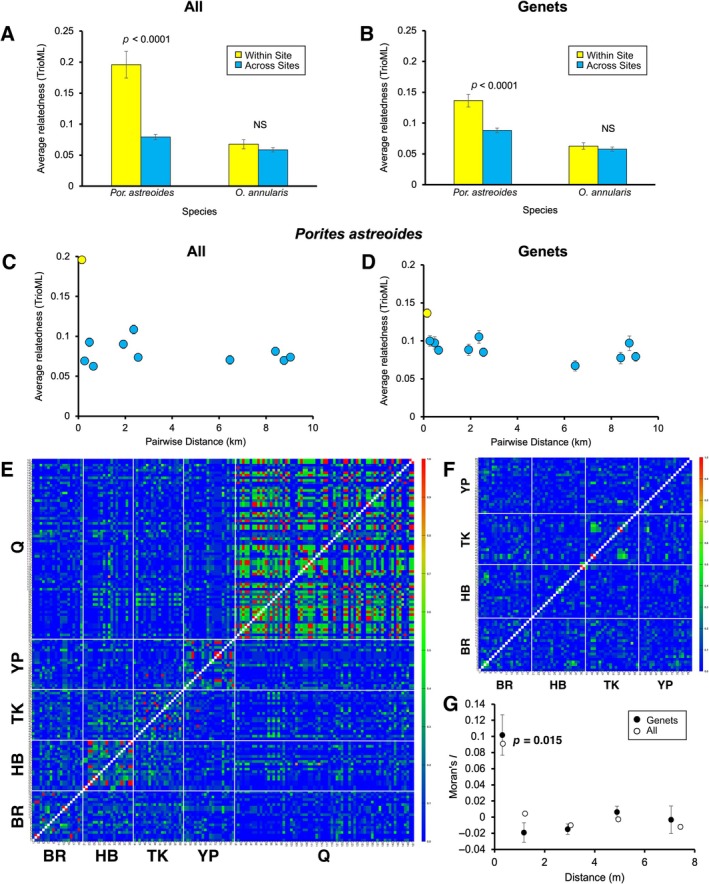
Patterns of relatedness for All colonies (A, C) and Genets (B, D) within and across sites for *Porites astreoides* and *Orbicella annularis*. Panels (C) and (D) are relatedness within and between sites as a function of pairwise site distance for *P. astreoides*. Significantly higher relatedness within sites was only noted in *P. astreoides*. Symmetrical heat maps of the spatial pattern of relatedness for *P. astreoides* (E) and *O. annularis* (F). Spatial autocorrelation of relatedness in *P. astreoides* at Site Q (G). All individuals (permutation test, *p* = 0.015) and a random sampling of ramets per genet (95% CI of 10 iterations did not include 0) were significantly different from random at only the closest distance class. NS, not significant; TrioML, triadic maximum likelihood estimator. BR, Boob Rock; HB, Hansen Bay; TK, Tektite; YP, Yawzi Point; Q, Site Q.

The simulations of relatedness found that for both species, means estimates of relatedness matched expectations (e.g., full‐sib/parent–offspring = 0.5, Appendix S1: Figure S3). The within‐reef populations of *P. astreoides* were largely composed of clonemates and related individuals (Figure 3). Average pairwise relatedness in *P. astreoides* was stronger within sites (*R* = 0.196 for “All” and 0.136 for “Genets”) than across sites (0.079 and 0.088 respectively, Figure 3A,B). The degree of relatedness within sites was higher in the analysis of All colonies compared with Genets as ramets within a genet have a value of 1.0. However, a significant pattern of higher within‐site relatedness remained in the analysis of “Genets” (All: *F*_1,13_ = 53.53, *p* < 0.0001; Genets: *F*_1,13_ = 29.57, *p* < 0.0001). The pattern of relatedness as a function of the pairwise distance between reefs indicated that the distinction of high relatedness within sites and low relatedness across sites occurs at even the most closely spaced reefs within Lameshur Bay (0.27 km, Figure 3C,D). This distinction of within‐site relatedness is present in Genets and is greatly magnified when considering asexual (parthenogenetic) propagation.

Site Q monitored for spawning had 71 genetically sampled colonies (out of the 184 total colonies within this site, Figure 3E, Appendix S1: Figure S5) and revealed 26 genets. Nine of these genets had multiple ramets with an average of 6.0 ramets per genet (SE = 1.7). Overall, the genets were related on average by 0.12, and across All colonies averaged 0.26 due to the prevalence of multiple ramets per genet. The number of ramets and relatives per genet is likely underestimated because only 38% (71 of 184) of colonies were sampled in this area. A spatial autocorrelation analysis of “All” individuals sampled within Site Q revealed a signal that individuals at the 0.5‐m distance class were more related to each other than expected by chance (permutation test *p* = 0.015, Figure 3G). A spatial autocorrelation of genets, in which one ramet per genet was randomly selected (iterated 10 times), showed the same pattern (95% CI of the 10 iterations did not include 0, Figure 3G). This suggests that within‐site spatial autocorrelation is driven by larval dispersal and survivorship rather than asexual fragmentation. There was a positive relationship between the maximum size of each genet and the number of ramets per genet (*R* ^2^ = 0.49, *F*_1,24_ = 22.9, *p* < 0.001, Appendix S1: Figure S7A). There was also a wide distribution of ramet sizes within a genet with a maximum range of 2–488 cm^2^ (Appendix S1: Figure S7B).

We detected no evidence of localized patterns of relatedness in *O. annularis*. The level of relatedness averaged around 0.06 and did not differ within and across sites for both All (*F*_1,8_ = 1.61, *p* = 0.23) and Genets (*F*_1,8_ = 0.69, *p* = 0.43) genotypes (Figure 3A,B). Overall, there was little evidence that *O. annularis* corals were related to each other regardless of whether they were within meters or kilometers of each other (Figure 3A,B,F).

### Inbreeding and genetic diversity

There was no significant difference in the mean inbreeding of genets (maximum likelihood estimate of *F*) between *P. astreoides* (0.122 SD = 0.12) and *O. annularis* (0.125 SD = 0.12), or the distribution of inbreeding among genets between these two species (*D* = 0.13, *p* = 0.44 Kolmogorov–Smirnov test). There was no significant difference in the level of genetic diversity (*H*_exp_) between species (*F*_1,7_ = 4.18, *p* = 0.0803, Appendix S1: Tables S1 and S2).

### Larval genetics

We obtained larvae from 13 of 18 colonies monitored in 2023. Of these 13 colonies, we detected 7 genets and genotyped 91 larvae (Appendix S1: Table S3) all of which shared the same genotype as the maternal colony. The program Genclone 2.0 indicated the probability (P_sex_ ranged from 0.0016 to <0.0001) that these larvae were the product of self or outcrossed fertilization was small. The low P_sex_ probabilities suggest these larvae were the product of parthenogenetic reproduction.

### Population dynamics

**FIGURE 4:**
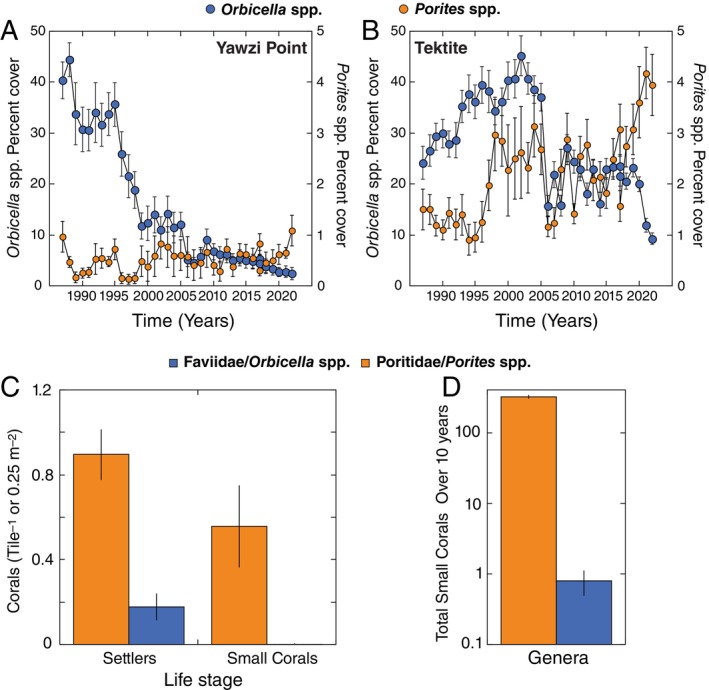
Long‐term population data. Mean percent cover (±SE) of *Orbicella* spp. and *Porites* spp. at Yawzi Point (A; *N* = 18–30 quadrats/year) and Tektite (B; *N* = 29–30 quadrats/year) from 1987 to 2022. Note the difference in scale for each genus. The density of settlers and small corals ≤4 cm diameter (C). Settlers identified to family level on tiles immersed for 1 year (15 tiles/site, mean for 5 sites [±SE]). Density of corals ≤4 cm identified to genus on natural substrata (40 quadrats/site, 6 sites averaged across 10 years from 2015 to 2024 ± SE). The number of small corals from panel (C), summed across 10 years (mean across sites, mean ± SE, note log scale; D).

The percent cover of *Orbicella* spp. and *Porites* spp. exhibited large changes over time at Yawzi Point and Tektite (Figure 4A,B). At Yawzi Point, the percent cover of *Orbicella* spp. declined (at 1.103%/year, *F*_1,35_ = 179.272, *p* < 0.001) from 40.4% ± 3.6% in 1987 to 2.5 ± 1.2% in 2022, while *Porites* spp. increased (at 0.009%/year, *F*_1,34_ = 8.535, *p* = 0.006) from 0.5% ± 0.1% (1988) (it was 1.0% ± 0.3% in 1987) to 1.1% ± 0.3% (2022). At Tektite, the percent cover of *Orbicella* spp. declined (at 0.531%/year, *F*_1,35_ = 20.830, *p* < 0.001) from 24.2% ± 3.3% in 1987 to 9.2% ± 4.0% in 2022, while *Porites* spp. increased (at 0.054%/year, *F*_1,35_ = 27.481, *p* < 0.001) from 1.5% ± 0.4% (1987) to 4.0% ± 0.6%. Although the absolute cover of *O. annularis* is greater than *P. astreoides*, shifts in cover within a species are more informative about demographic changes than comparisons of absolute cover between species that differ in colony size. *O. annularis* grows via the proliferation of lobe‐shaped ramets each averaging ca. 30 cm in diameter that together can extend for several meters (Edmunds, 2019), while *P. astreoides* colonies average ca. 2–4 cm in diameter (Edmunds et al., 2021) and rarely exceed 15 cm (Appendix S1: Figure S7B).

The abundances of early life stages of *Porites* and *Orbicella* also show striking differences between taxa. Poritid settlers were 5‐fold more abundant than faviids on settlement tiles, and small colonies of *Porites* were 180‐fold more abundant than small colonies of *Orbicella* on natural reef surfaces (Figure 4C). The discrepancy in abundance is underscored by the 397‐fold greater number of small colonies of *Porites* encountered during annual surveys compared with small colonies of *Orbicella* (Figure 4D). Analyses of the mortality rates of small colonies also highlight the differences between genera, with *Porites* dying at a mean (±SE) rate of 29% ± 4%/year (*n* = 370 colonies over 10 years), and a small number of replicate colonies of *Orbicella* suggesting they died at a rate of 10% ± 7%/year.

## DISCUSSION

A brooding rather than broadcasting reproductive strategy enhances fertilization success at low population densities (Yund, 2000), increases nutritional provisioning of each offspring (Emlet & Hoegh‐Guldberg, 1997), decreases both the planktonic larval period and dispersal away from parental habitats (Carlon & Olson, 1993; Hellberg, 1996) and is associated with releasing larvae many times during a season (Soong, 1991). These traits all have been suggested to contribute to a life history strategy promoting enhanced persistence in disturbed habitats where unoccupied space is unpredictably available (Goodbody‐Gringley, 2016). This notion is highlighted in the current comparison of *P. astreoides* and *O. annularis*, where higher settlement success of *Porites* leads to a two‐orders‐of‐magnitude difference in the abundance of corals <4 cm in size, and over 36 years has favored long‐term increases in cover of *Porites* spp., while *Orbicella* spp. has declined. This provides insight into the demographic success of *P. astreoides* compared with *O. annularis*; decades of environmental stress have increased the mortality of adult corals (Hughes et al., 2017; Rogers, 2009) and species that can quickly recruit into ephemeral open space will have the advantage (Arnold et al., 2010; Goodbody‐Gringley, 2016). In St. John and at other locations in the Caribbean (Green et al., 2008), *P. astreoides* has been successful on shallow reefs in recent decades. Prior studies from Yawzi Point (2–6 m depth) where most corals recently have been recruiting to open space created by grazing sea urchins (Levitan et al., 2023) belong to brooding species (98%) but of these brooders, most were *Porites* (86%) and the majority of *Porites* were *P. astreoides* (62%). The trait that might distinguish *Porites* spp. (Lord et al., 2023) and specifically *P. astreoides* from other brooding corals is the capacity for parthenogenetic reproduction. Parthenogenesis, combined with brooding, leads to the proliferation and retention of successful genotypes at a scale of 50–100 m, without the costs associated with inbreeding.

### Population structure and clonal propagation

We found genetic structuring for *P. astreoides* at fine spatial scales, enhanced by high rates of parthenogenic larval propagation. Across the Caribbean, *P. astreoides* clonality has been estimated on average to be 18.3%, but it varies between 0% and 50% among sites (Riquet et al., 2022). Among colonies in the present study, we found an average of 28.8% clonality in our population genetic survey across four sites that increased to 59.2% within our finer scale sampling within a 50‐m^2^ plot. It is not surprising that as the sampling effort increased, the number of colonies with clonemates increased. The prevalence of clones within sites is likely due to parthenogenesis as opposed to fragmentation of adult colonies, as evident by the production of parthenogenetic larvae and the ~1–50 m distance between clonemates.

Previous population genetic studies of *P. astreoides* using genets (one representative ramet per genet) have reported genetic structure at the regional scale in the Caribbean (Riquet et al., 2022, but see Serrano et al., 2016). Within the Lesser Antilles, *P. astreoides* demonstrates significant isolation by distance. Around Guadeloupe, values of *F*_ST_ were similar across sites separated by 9 km and within sites separated by ~25 m depth (*F*_ST_ averaging ~0.04 for both comparisons, Riquet et al., 2022). Depth‐related genetic isolation was also noted in Florida but not Bermuda or the US Virgin Islands (Serrano et al., 2016). Spatially, the within‐island values of *F*_ST_ for *P. astreoides* observed in Guadeloupe (Riquet et al., 2022) are similar to the present values of genets in St. John. In our study, the *F*_ST_ values are nearly doubled when considering the influence of asexual reproduction through parthenogenesis. Incorporating the influence of parthenogenesis revealed genetic structure at all pairwise site distances, even sites 0.27–0.47 km apart within Greater Lameshur Bay (Figure 2).

We suggest that comparing population genetics of Genets versus All colonies is revealing as it highlights the contribution of asexual reproduction. This suggestion is supported by simulation studies of how clone correction influences genetic summary statistics (Meirmans, 2024). Meirmans (2024) advocates for this comparative approach and cautions against using a genet‐only approach. Generally, the genet‐only approach is used to estimate population structure in corals (e.g., Riquet et al., 2022; Severance & Karl, 2006). The rationale is that in most cases clonal propagation of corals is generated by colony growth followed by fission and fragmentation from a single settling sexual larva. Storms can break apart even massive corals, such as *Orbicella*, and disperse colony fragments several meters (Foster et al., 2013). However, colony fragmentation is unlikely to contribute to genetic mixing across sites. In contrast, brooded parthenogenetic larvae have, in principle, the same dispersive ability and the same capacity to generate or limit population structure as brooded sexual larvae. Although we rarely detected clonemates of *P. astreoides* across reefs in St. John, other studies of *P. astreoides* along the coast of Florida found clonemates separated by 50 km (Shilling et al., 2023). Given the high degree of relatedness within reefs and low degree of relatedness across closely spaced reefs, independent of clonal propagation (Figure 3), the rarity of finding clonemates across reefs might reflect overall high levels of larval retention within reefs. Genetic structure enhanced by parthenogenesis is an important feature of *P. astreoides* and contributes to the distinctive genetic identity of each reef.

In contrast, *O. annularis* in St. John exhibited little evidence of population structure. This is likely due to the broadcast spawning life history strategy that results in dispersing gametes and larvae (Levitan et al., 2004). Previous studies of *O. annularis* have shown significant genetic structure at a regional spatial scale in the Caribbean (Foster et al., 2013; Severance & Karl, 2006), but like our study, found little genetic differentiation and no evidence of isolation by distance at smaller spatial scales (Severance & Karl, 2006). Additionally, clonality has at times been reported to be high for *O. annularis* (18.6%), particularly when correlated with hurricane events, indicating that clonality is driven by asexual reproduction through fragmentation (Severance & Karl, 2006). We found low levels of clonality in *O. annularis*, perhaps reflecting our sampling scheme of mixing nearest neighbors with pairs isolated by 5 m. Prior genetic work in Lameshur Bay found *O. annularis* clonemates clustered at the meter scale (Edmunds et al., 2014). Unlike *P. astreoides*, for *O. annularis*, genet analysis might be a more appropriate measure of the degree of genetic isolation among sites. However, in both species, incorporating asexual propagation reveals insight into what mechanism contributes to the distribution of genes and genotypes within and across reefs.

For *P. astreoides*, the combination of a brooding and parthenogenesis leads to reefs consisting of groups of clonemates and their relatives. In our study, estimates of relatedness were based on genotypes at eight and nine markers in *P. astreoides* and *O. annularis*, respectively. Simulations indicated the marker panels of both species had comparable resolution and ability to quantify relatedness from genotypic states (Appendix S1: Figure S3). Nonetheless, previous work has demonstrated the limitations of estimating relatedness with relatively small marker panels (Attard et al., 2018; D'Aloia et al., 2018), and we suggest using caution in interpreting genetic estimates of relatedness as realized relationship types (e.g., parent–offspring and full siblings). The average level of relatedness across reefs (0.08–0.09 for All and Genets) was similar to that found within and across reefs in *O. annularis* (0.06–0.07). In contrast, the within‐site estimate of relatedness for *P. astreoides*, even for reefs separated by <300 m, was 0.20 and 0.14 for All and Genets, respectively. Site Q, with 71 colonies of *P. astreoides* sampled within a 50 m^2^, had the highest resolution of population structure. The combination of a high degree of relatedness within this site (*R* = 0.26) compared with its relation to a site <300 m away (Yawzi Point, *R* = 0.07) and the higher within‐site relatedness at a scale of 1 m^2^ suggests a signal of high larval retention of sexual and asexual larvae at scales of ~50 m. This matches prior work on the related parthenogenetic congener, *P. divaricata*, which concluded that dispersal in this brooding species was typically limited to 50–100 m (Lord et al., 2023). Retention of larvae, as opposed to the collective dispersal of clonal larvae from a more distant source, seems more likely given the wide size distribution of clonemates within a reef (Appendix S1: Figure S7B).

### Inbreeding, genetic diversity and local adaptation

Estimates of inbreeding and genetic diversity of genets were similar for *P. astreoides* and *O. annularis* and did not differ from predictions of random mating. This pattern was surprising given the elevated relatedness among *P. astreoides* colonies within sites and the spatial autocorrelation of related individuals at the scale of ~1 m. The absence of inbreeding in *P. astreoides* suggests that close relatives rarely mate or perhaps avoid mating. Brooding ascidians can block self‐sperm and discriminate sperm from competing outcrossed males (Bishop et al., 1996), so the potential for brooding species to avoid inbreeding is possible. Although we did not detect sexual reproduction in brooded larvae of *P. astreoides*, the finding of a high relatedness among individuals within sites suggests that sexual reproduction is occurring, and prior work has documented outcrossing in this species (Brazeau et al., 1998, but see Vollmer, 2018). The finding of sexual and parthenogenetic reproduction was also found in the brooding Pacific coral *Pocillopora damicornis* (Ayre & Miller, 2004; Combosch & Vollmer, 2013). Ayre and Miller (2004) found that larval broods were 100% parthenogenetic and concluded that this conflicted with the high levels of genotype diversity they observed. Combosch and Vollmer (2013) found that larval broods were predominately parthenogenetic in this species, but small colonies (<100‐cm^2^ area) were more likely to also produce sexual larvae. They concluded that this mixed reproductive strategy could propagate successful genotypes while maintaining genetic diversity. Our study indicates that primarily parthenogenetic reproduction along with high levels of larval retention within sites, mixed with occasional sexual reproduction and periodic gene flow can proliferate locally successful genotypes while avoiding inbreeding and maintaining genetic diversity. We also found that the largest *P. astreoides* genets had the greatest number of ramets; genets surviving to a large size disproportionately populated these reefs.

Evidence for local adaptation has been noted in *P. astreoides*. Transplant experiments have revealed immune‐related gene expression differences among inshore and offshore populations (Haslun et al., 2021), and a genetic basis for among‐site variation in growth rate with local genotypes outperforming transplanted genotypes (Kenkel et al., 2015). For *P. astreoides*, we hypothesize that brooding favors the local retention of larvae, and parthenogenesis keeps locally successful genotypes intact (free from recombination), at least when “success” is defined as survival to reproductive maturity. Together these traits might explain the observation of local adaptation in *P. astreoides*.

Restoration strategies for corals now consider the use of assisted evolution targeting genotypes with increased stress tolerance (van Oppen et al., 2014). Artificially selecting, or creating, stress‐tolerant genotypes in the laboratory might mirror what *Porites* has undergone through decades of natural selection on reefs exposed to a wide variety of frequently occurring disturbances. The current study suggests for *P. astreoides* that brooding coupled with parthenogenetic reproduction might have facilitated local persistence over decades of overall declining coral cover. The mixture of occasional sexual reproduction and periodic dispersal to more distant reefs might maintain sufficient genetic diversity such that the asexual proliferation of multiple genets, selected by nature, outweighs the loss of some random or poorly suited genets when conditions change. A parthenogenetic strategy contributes to dandelions being aggressively invasive in disturbed terrestrial habitats (e.g., Molina‐Montenegro et al., 2013) and might forecast which coral species persist in the face of degrading reefs in the marine realm.

## AUTHOR CONTRIBUTIONS

Don R. Levitan conceptualized the study and contributed to the design, data collection, and analysis and was primarily responsible for the writing. Kevin C. Olsen analyzed the genetic data and contributed to the writing. Rachael M. Best contributed to the fieldwork and writing. Peter J. Edmunds contributed to the design, data collection, analysis of demographic data, and writing.

## CONFLICT OF INTEREST STATEMENT

The authors declare no conflicts of interest.

## Supporting information


Appendix S1.

## Data Availability

Data (Levitan et al., 2025) are available in Dryad at https://doi.org/10.5061/dryad.pg4f4qrz4.
