# Supplementary material for: Brooding and parthenogenesis enhance the success of the coral Porites astreoides relative to Orbicella annularis
Source: Ecology. 2025 May 8;106(5):e70102. doi: 10.1002/ecy.70102 (PMC12062785; doi:10.1002/ecy.70102)
Supplement: Supplementary file 1 — Appendix S1. [file ECY-106-e70102-s001.pdf]

Appendix S1

Don R. Levitan, Kevin C. Olsen, Rachael M. Best and Peter J. Edmunds

Brooding and parthenogenesis enhance the success of the coral *Porites astreoides* relative to *Orbicella annularis*

Journal Name: Ecology

Figure S1. Photograph of larval trap placed over a *P. astreoides* colony. Traps were deployed in the late afternoon each day of sampling. Larval release occurs at night (McGuire 1997) travel upwards and get caught in the trap. In the morning, the conical container was detached from the plastic container, capped and brought to the lab and larvae were fixed for genetic analysis. Photo credit: Rachael M. Best.

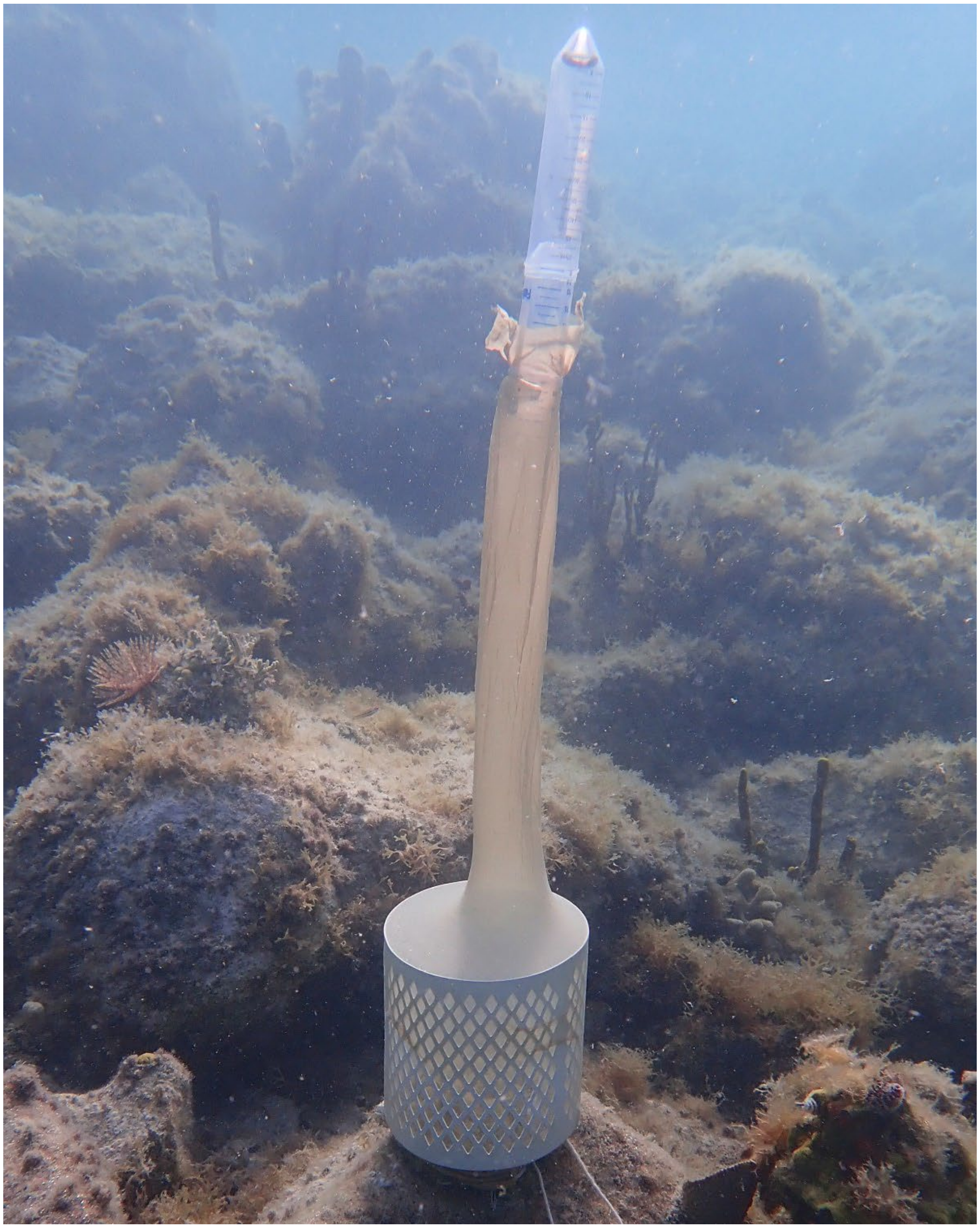

Figure S2. Genotype accumulation curves implemented with the *P. astreoides* and *O. annularis* marker sets. These simulations quantify the power to delineate multi-locus genotypes (MLG), and indicate that six and four loci were sufficient to identify clonal and non-clonal members within *P. astreoides* and *O. annularis*, respectively. In practice, we evaluated eight and nine loci in the *P. astreoides* and *O. annularis* marker sets, suggesting our analyses had sufficient power to distinguish clonal and non-clonal genotypes within both species.

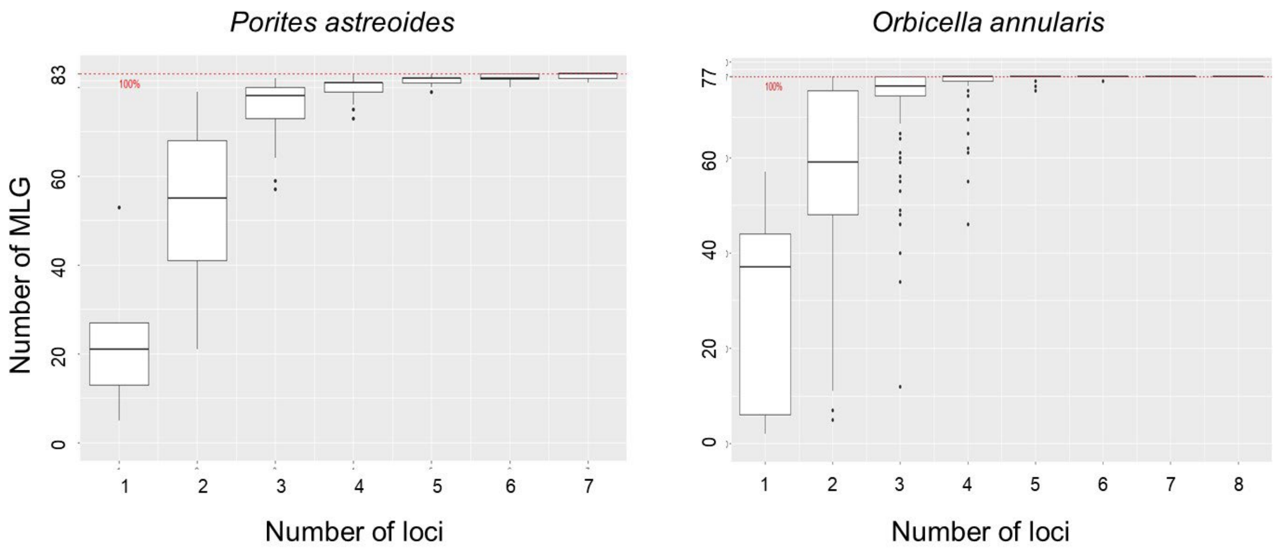

Figure S3. Pairwise estimates of relatedness (TrioML) implemented on simulated genotypes. Genotypes were simulated in the program Coancestry (Wang 2011), using the characteristics of each marker set (**A** *P. astreoides*; **B** *O. annularis*), including number of loci, degree of polymorphism, and allele frequency. For each class of simulated relationship, we compared estimates of relatedness to values expected under random mating (i.e., 0.5 for parent-offspring, 0.5 for full siblings, 0.25 for half-siblings, 0.125 for first cousins, and 0 for unrelated pairs). Because of the variance within each category and the relatively small marker panel size, assigning empirical values to specific relatedness categories should be made with caution (Attard et al. 2018, D'Aloia et al. 2018).

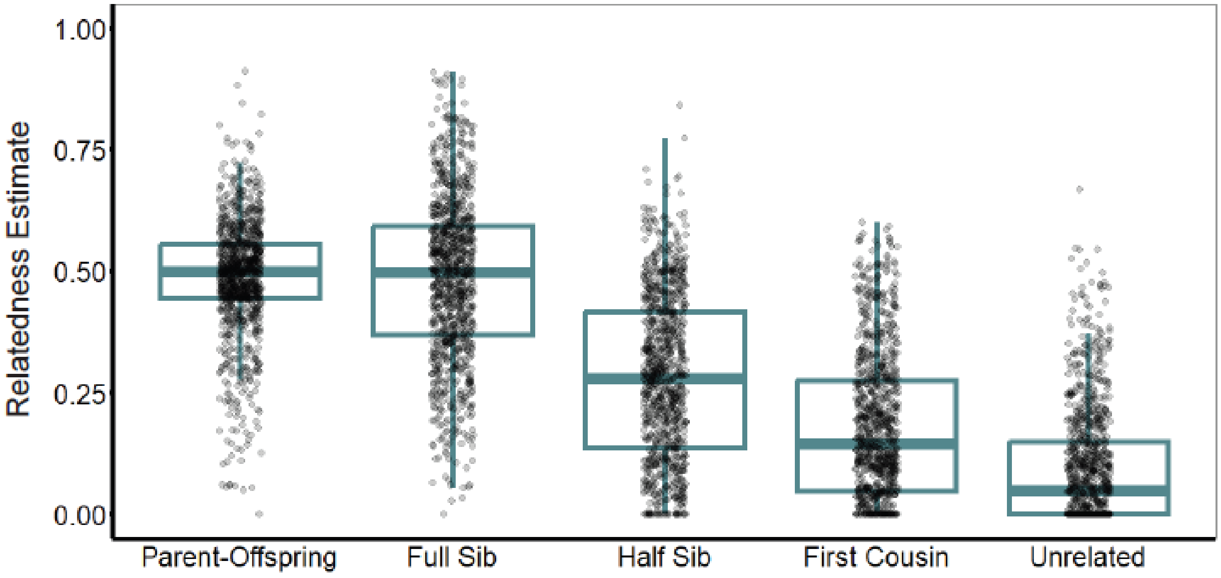

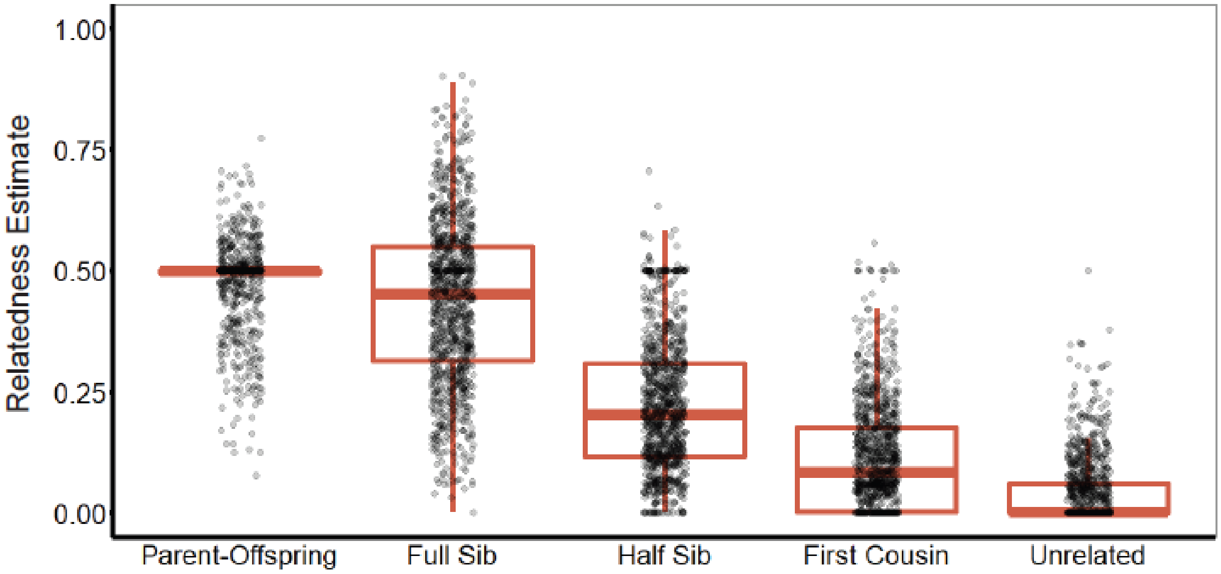

Figure S4. Distribution of sampled *Porites astreoides* colonies at four sites along the south shore of St. John, US Virgin Islands. In this figure, clone-mates within a site share colors; instances of clone-mates across reefs are indicated by asterisks.

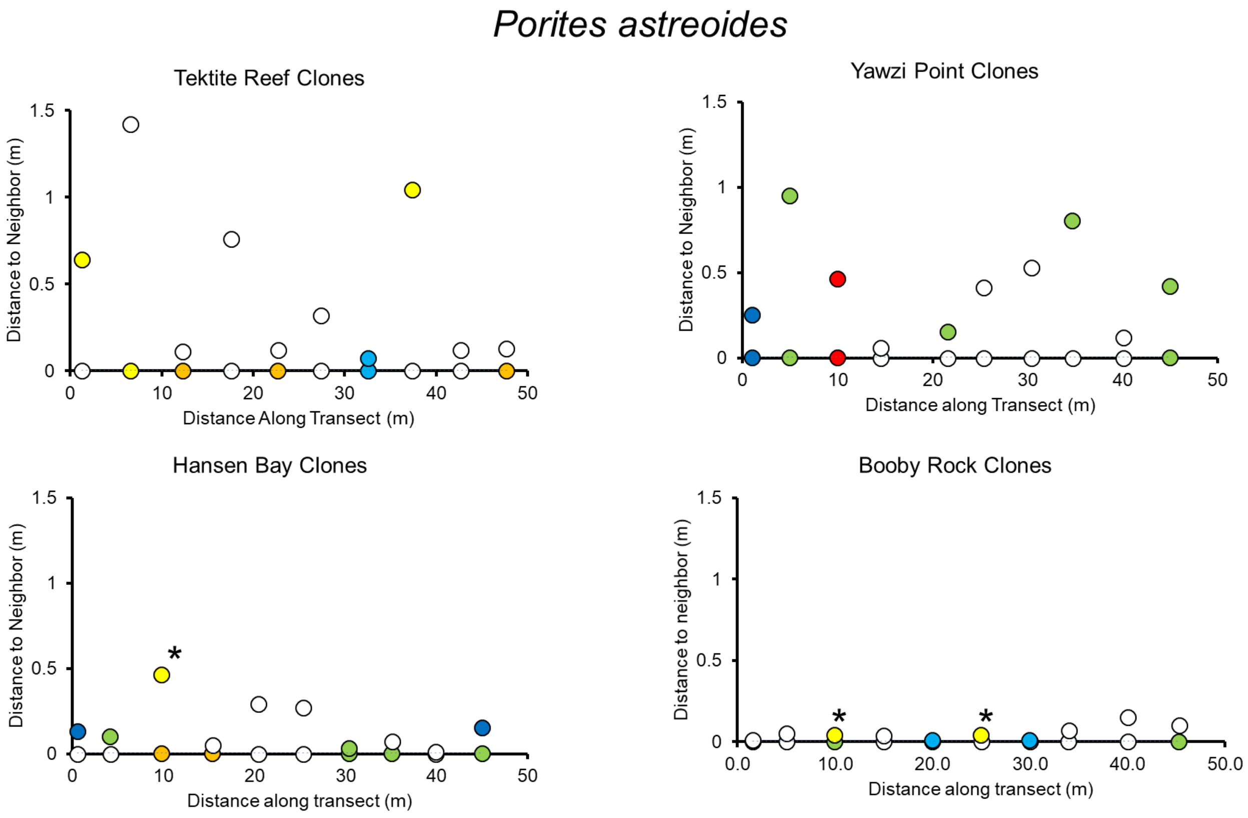

Figure S5. Distribution of *P. astreoides* at site Q used to collect larvae of *P. astreoides*. This site was ~ 10 x 5 m and was subdivided into 1m^2 quadrats to facilitate mapping of individual corals to genotype. In this figure, clone-mates share a common color, and open symbols are unique MLG. Maternal colonies are identified by a box around the symbol. Genetic samples and colony size were obtained for all colonies within the same 1 m^2 as each maternal colony. Small black symbols are unsampled *P. astreoides* in the site but outside of the 1m^2 quadrats occupied by maternal colonies, these individuals were mapped to access the density of *P. astreoides* at this site.

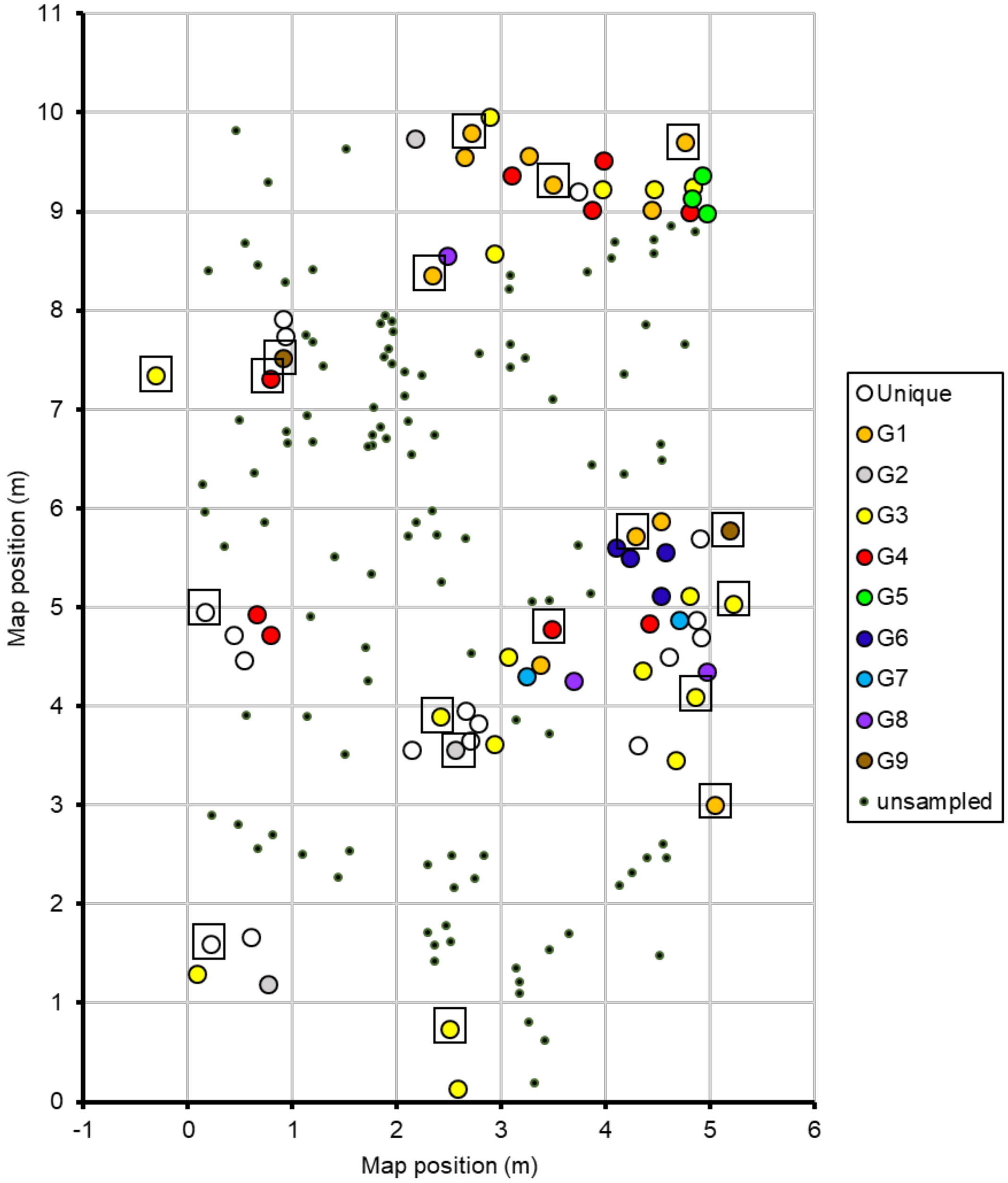

Figure S6. Distribution of sampled *Orbicella annularis* colonies at four sites along the south shore of St. John, US Virgin Islands. In this figure clone-mates within a site share a common color.

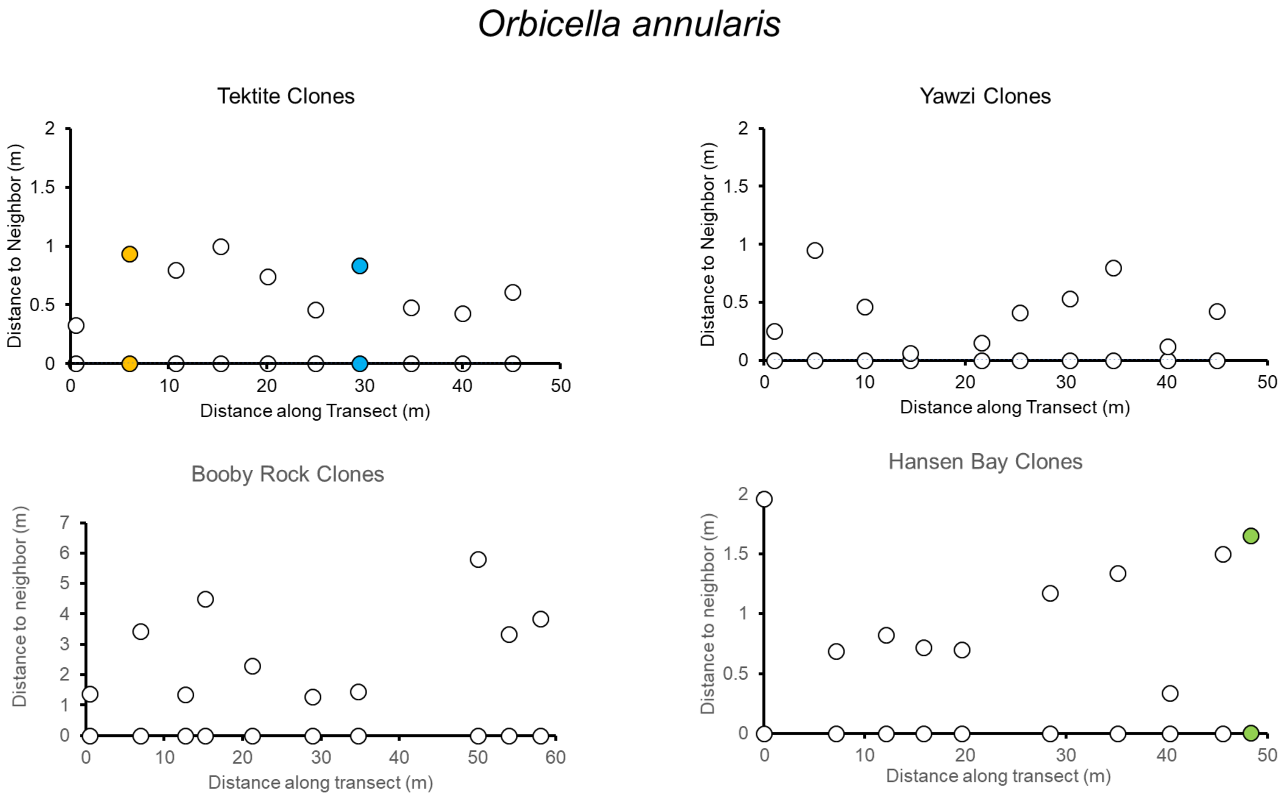

Figure S7. Colony sizes (cm2) of *P. astreoides* at site Q. A. The number of ramets per genet as a function of the largest ramet (coral colony) of each genet. The number of ramets increased with the size of the largest ramet ($F_{1,24} = 22.9$, $P < 0.001$). B. The distribution of ramet coral sizes within each genet. Genet ID listed in legend, Genets with only one ramet found have a clear symbol (Unique).

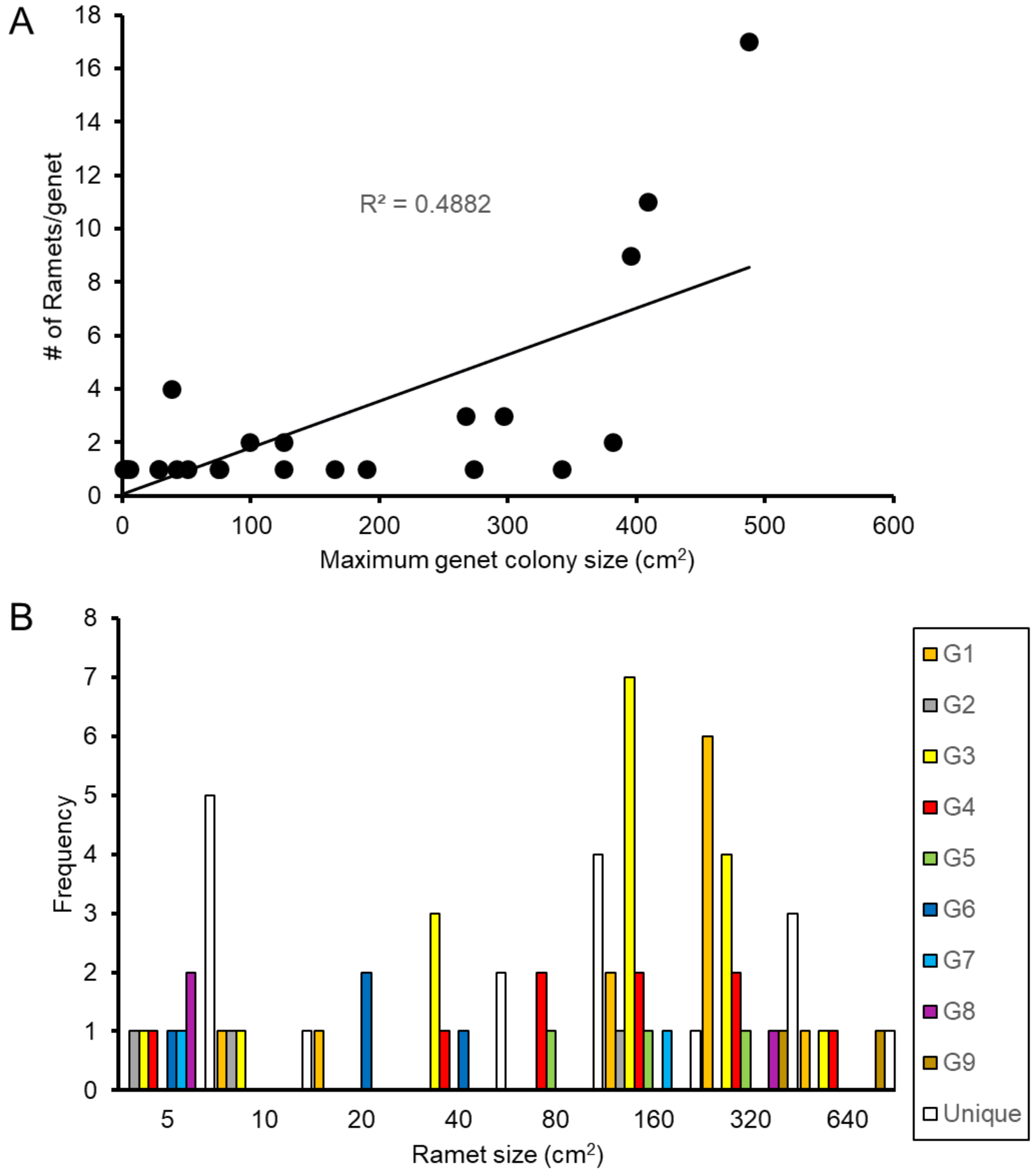

Table S1. Genetic summary of microsatellite data for *P. astreoides* of genets (unique MLG). References for loci details; 1 (Serrano et al. 2016), 2 (Kenkel et al. 2013).

**Locus Summary**					
**Locus**	**Alleles**	**Heterozygosity Observed**	**Heterozygosity Expected**	**FIS**	**Reference**
PA69	6	0.59	0.53	-0.107	1
Past8	12	0.59	0.64	0.077	2
Past17	9	0.78	0.78	-0.001	2
Past13	10	0.49	0.72	0.328	2
PA7	3	0.37	0.35	-0.046	1
Past10	21	0.86	0.91	0.058	2
Past16	6	0.73	0.69	-0.056	2
Past3	8	0.70	0.73	0.048	2
**Average**				**0.038**	
**Population Summary**					
**Population**	**N**	**MLG**	**Heterozygosity Observed**	**Heterozygosity Expected**	**FIS**
TC	20	15	0.60	0.68	0.120
YP	20	13	0.52	0.64	0.169
BR	20	16	0.67	0.66	-0.048
HB	20	14	0.72	0.71	-0.023
Q	71	26	0.67	0.65	-0.008
**Total**	151	83			
**Average**					**0.042**

Table S2. Genetic summary of microsatellite data for *O. annularis* of genets (unique MLG). References for loci details; 1 (Severance et al. 2004), 2 (Davies et al. 2013).

**Locus Summary**					
**Locus**	**Alleles**	**Heterozygosity Observed**	**Heterozygosity Expected**	**FIS**	**Reference**
maMS8	3	0.10	0.12	0.160	1
Mfav8	19	0.82	0.91	0.100	2
Mfav3	5	0.41	0.38	-0.086	2
Mfav5	14	0.78	0.77	-0.004	2
maMS2-8	15	0.84	0.83	-0.016	1
Mfav9	19	0.85	0.89	0.037	2
Mfav30	2	0.01	0.01	0.000	2
Mfav29	15	0.83	0.88	0.058	2
maMS11	33	0.61	0.94	0.353	1
**Average**				**0.067**	
**Population Summary**					
**Population**	**N**	**MLG**	**Heterozygosity Observed**	**Heterozygosity Expected**	**FIS**
BR	20	20	0.55	0.64	0.215
HB	20	19	0.57	0.62	0.043
TC	20	18	0.59	0.64	0.067
YP	20	20	0.63	0.65	0.000
**Total**	80	77			
**Average**					**0.081**

Table S3. Distribution of genotyped coral larvae from *P. astreoides* as a function of collection date, maternal colony ID and genotype. The Full Moon was on 1 August 2023. Colonies marked with a common color are ramets in the same genet, colonies without color are unique genets. All genotyped larvae were an exact match to their maternal colony.

Coral ID	Jul. 30	Jul. 31	Aug. 1	Aug. 2	Aug. 3	Aug. 4	Aug. 5	Aug. 6	Aug. 7	Aug. 8	Aug. 9	Aug. 10	Aug. 11	**Sum**
220														**0**
221				1										**1**
222												4		**4**
223										11				**11**
224					9	3								**12**
225											1	1		**2**
226														**0**
227														**0**
228														**0**
229		9		4										**13**
230														**0**
231											1			**1**
232				12					1					**13**
233												1		**1**
234								1						**1**
235						4	1		7					**12**
236										11				**11**
237						1	4				4			**9**
**Sum**	**0**	**9**	**0**	**17**	**9**	**8**	**5**	**1**	**8**	**22**	**6**	**6**	**0**	**91**

References

Attard, C. R. M., L. B. Beheregaray, L. M. Moller. 2018. Genotyping-by-sequencing for estimating relatedness in nonmodel organisms: avoiding the trap of precise bias. *Molecular Ecology Resources* 18:381–390.

D'Aloia, C. C., A. Xuereb, M. J. Fortin, S. M. Bogdanowicz, and P. M. Buston. 2018. Limited dispersal explains the spatial distribution of siblings in a reef fish population. *Marine Ecology Progress Series* **607**, 143–154.

Davies, S.W., M Rahman, E Meyer, E.A. Green, E Buschiazzo, M. Medina, M. Matz. 2013. Novel polymorphic microsatellite markers for population genetics of the endangered Caribbean star coral, *Montastraea faveolata*. *Marine Biodiversity*. 43:167-172.

Kenkel, C.D., G. Goodbody-Gringley, D. Caillaud, S.W. Davies, E. Bartels, M.V. Matz. 2013. Evidence for a host role in thermotolerance divergence between populations of the mustard hill coral (*Porites astreoides*) from different reef environments. *Molecular Ecology* 22:4335-4348.

McGuire, M. P. 1997. The biology of the coral *Porites astreoides*: Reproduction, larval settlement behavior and responses to ammonium enrichment. Dissertation. University of Miami, Miami, Florida, USA.

Serrano, X. M., I. B. Baums, T. B. Smith, R. J. Jones, T. L. Shearer, and A. C. Baker. 2016. Long distance dispersal and vertical gene flow in the Caribbean brooding coral *Porites astreoides*. *Scientific reports* **6**:21619.

Severance, E.G., A.M. Szmant, S.A. Karl. 2004. Microsatellite loci isolated from the coral, *Montastraea annularis*. *Molecular Ecology Notes* 4:74-76.

Wang, J. C. 2011. COANCESTRY: A program for simulating, estimating and analysing relatedness and inbreeding coefficients. *Molecular Ecology Resources* **11**:141–145.
